# Current Insights on Antifungal Therapy: Novel Nanotechnology Approaches for Drug Delivery Systems and New Drugs from Natural Sources

**DOI:** 10.3390/ph13090248

**Published:** 2020-09-15

**Authors:** Filipa Sousa, Domingos Ferreira, Salette Reis, Paulo Costa

**Affiliations:** 1 UCIBIO, REQUIMTE, Laboratory of Pharmaceutical Technology, Department of Drug Sciences, Faculty of Pharmacy, University of Porto, Rua Jorge Viterbo Ferreira nº 228, 4050-313 Porto, Portugal; domingos@ff.up.pt; 2 LAQV, REQUIMTE, Department of Chemical Sciences, Faculty of Pharmacy, University of Porto, Rua Jorge Viterbo Ferreira nº 228, 4050-313 Porto, Portugal; shreis@ff.up.pt

**Keywords:** nanoparticles, fungi, drug delivery systems, marine, biological synthesis, myconanotechnology

## Abstract

The high incidence of fungal infections has become a worrisome public health issue, having been aggravated by an increase in host predisposition factors. Despite all the drugs available on the market to treat these diseases, their efficiency is questionable, and their side effects cannot be neglected. Bearing that in mind, it is of upmost importance to synthetize new and innovative carriers for these medicines not only to fight emerging fungal infections but also to avert the increase in drug-resistant strains. Although it has revealed to be a difficult job, new nano-based drug delivery systems and even new cellular targets and compounds with antifungal potential are now being investigated. This article will provide a summary of the state-of-the-art strategies that have been studied in order to improve antifungal therapy and reduce adverse effects of conventional drugs. The bidirectional relationship between Mycology and Nanotechnology will be also explained. Furthermore, the article will focus on new compounds from the marine environment which have a proven antifungal potential and may act as platforms to discover drug-like characteristics, highlighting the challenges of the translation of these natural compounds into the clinical pipeline.

## 1. Introduction

There is a wide range of fungal infections, from superficial, affecting skin, to systemic infections with invasion of internal organs [1]. Fungal infections affect millions of people every year worldwide. Of these, more or less 1.5 million are invasive fungal infections therefore requiring advanced treatment and hospitalization. Most of these disseminated infections are caused by *Candida, Cryptococcus, Aspergillus,* and *Pneumocystis* species, being the cause of cryptococcosis, candidiasis, aspergillosis, and pneumocystis pneumonia, respectively [2].

Superficial fungal infections are rather common and, despite rarely being life threatening, they can spread to other skin regions and even become widespread. Furthermore, they can be transmitted to other people and may cause secondary bacterial skin infections, harming the quality of a person’s life. Skin mycoses are classified according to the causative fungal agents into dermatophytosis, yeast infections, and mold infections [1].

Invasive fungal infections represent a significant burden to healthcare systems, having high morbidity and mortality rates. These rates are most worrisome among immunocompromised patients that are more prone to opportunistic infections, such as patients with Acquired Immune Deficiency Syndrome (AIDS), transplant patients whose immune systems are suppressed to prevent organ rejection, patients with cancer who are taking immunosuppressive chemotherapy or autoimmune patients undergoing immunosuppressive therapy [2,3].

The currently major available agents to treat invasive fungal infections can be grouped into four main classes according to their mechanism of action: polyenes, azoles, allylamines, and echinocandins (Table 1) [4]. They all present drawbacks when it comes to spectrum of activity, drug–drug interactions, pharmacokinetics and pharmacodynamics, resistance mechanisms, and the toxicity of the compounds themselves. Furthermore, there are some limitations in terms of clinical efficacy and efficiency, mainly because of their physical-chemical properties, like their hydrophobic character that leads to a low solubility in water and also selectivity problems deriving from the similarities between fungi and human cells [3,5].

Nevertheless, the design and development of new drug delivery systems or even new antifungals is an emerging need, owing to the following facts [8]:

- There are 20–40% mortality rates with invasive mycoses, therefore these figures need to be improved;
- The increase in patients undergoing prolonged antifungal therapies reflects the need to develop better fungicidal drugs and thus reduce the length of the treatments and the costs associated;
- There is still space for improvement in pharmacokinetics and pharmacodynamics, in order to reduce the frequency of drug use;
- More attention needs to be given to the host toxicities and drug–drug interactions of current therapy so that their effects can be eliminated or, at least, minimized;
- New therapy groups with different mechanisms of action are needed; this way, these new drugs may synergize with present ones and allow better responses;
- There is an alarming growth in antifungal resistance in all therapeutic groups [8].

**Figure 1:**
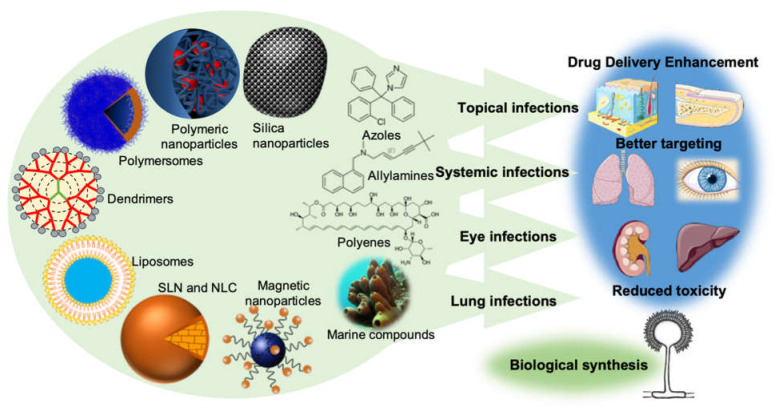
The new drug delivery systems based on nanotechnology that are currently being employed in order to enhance drug delivery, promote a better targeting, and reduce the toxicity of conventional antifungal drugs. It is also important to point out the importance of the production of nanoparticles by fungi (biological synthesis) and the undeniable potential of the sea as a source of new molecules with antifungal activity.

Nanotechnology is an emerging field of science that has shown an undeniable versatility and has boosted a revolution when it comes to medical treatments, quicker diagnosis, cellular regeneration, and drug delivery [9,10]. The material to produce nanoparticles can be divided into three main groups: polymers, lipids, or metals, each one giving rise to a different type of nanoparticle [11]. The main representatives of each of these three different groups of nanoparticles are mentioned in Figure 1 below.

Nanoparticles have been employed in pharmaceutical formulations because of their ability to alter and improve the pharmacokinetic and pharmacodynamic properties of the drugs. This is given to their capability to increase the solubility and stability of the drugs, to allow a controlled release and to exhibit biocompatibility with tissues and cells, which is reflected in an overall improvement on therapeutic efficiency [11,12]. In addition, its subcellular size is compatible with an intravascular injection and its high surface area is amenable to modification so that the drug is released in a specific target, thus reducing the systemic adverse effects and increasing the therapeutic compliance, by decreasing the usual dose and the frequency of administration [13,14]. This targeted-specific action is possible since, at a nanomolecular level, it is possible to incorporate target ligands that allow a preferential binding of certain types of cells, by conjugation with antibodies and peptides on the surface of the transporters [15,16,17]. Hence, the development of new biopharmaceutical systems, especially nanoparticulate carriers, is a good strategy to improve the therapeutic efficacy, safety, and compliance of conventional antifungal drugs.

In Table 2 an overview of the new antifungal drug delivery systems is presented, and the drug chemical group, their route of administration, and their dosage form provided.

However, the efficacy and human safety of these new therapies remain uncertain in most of the articles found in literature. They generally lack controlled clinical trials and sometimes the suggested routes of administration are less practical, or the production cost may hinder the replacement of the conventional treatment. Nevertheless, in other cases, the opposite is verified, and some options have potential to become a viable first line treatment [102]. Moreover, given the widespread use of antifungal agents and the limited therapeutic offer, fungi have developed resistance mechanisms, like overexpression of efflux pump proteins and formation of biofilms. These mechanisms can mean not only a decrease in a drug’s effective concentration, but also changes and subexpression of drug targets and metabolic bypass [6]. It is important to add that resistance is a cross-cutting issue to all of the currently available classes of antifungal agents, therefore overcoming antifungal resistance can be considered as the mainstay for improving therapeutic strategies to treat antifungal infections [2,103].

Despite the uprising of these issues in antifungal therapy, there are several mechanisms by which nanoparticles overcome the development of resistance mechanisms:

- The chemical features and simultaneous multiple mechanisms used by nitric oxide, chitosan, and metallic nanoparticles make the likelihood of resistance development unviable (for example, through the direct reaction of reactive nitrogen oxide intermediates with DNA structure) [104,105];
- The resistance mechanisms can be prevented by packaging multiple antimicrobial drugs within the same nanoparticle, because the likelihood of multiple simultaneous gene mutations in the same cell is low. The most striking examples are the encapsulation of antifungal drugs in chitosan or silver nanoparticles, combining the antifungal properties of both and decreasing the possibility of drug resistance [104,106];
- Some nanoparticles, such as liposomes and dendrimers, are able to overcome the resistance mechanisms of decreased uptake and increased efflux of drug from the microbial cell. Liposomes are able to quickly fuse with the plasma membrane of the microbial cell and release a high concentration of drug into its plasma membrane or cytoplasm, thereby circumventing the decreased uptake mechanism of resistance. This means a faster delivery and avoidance of the transmembrane pumps that catalyze increased efflux of drugs. Dendrimers, on the other hand, are extensively branched molecules, whose surface can be filled with positively charged quaternary ammonium compounds, which bind to negatively charged microbial cell envelopes and increase membrane permeability. This allows the entrance of more dendrimers to the microbial cell, the flow of its cytoplasmic contents to the exterior, and the ultimate destruction of the microbial cell membrane. This goes to show that dendrimers are also able to surpass the resistance mechanism of decreased uptake of drug [107]. Other nanoparticles, specifically nitric oxide nanoparticles made of silica and zinc oxide nanoparticles are able to overcome biofilm formation by killing the microbes present in already formed biofilms or by inhibiting biofilm formation through the generation of reactive oxygen species, respectively [108,109];
- Nanoparticles have been used to target antifungal drugs to the specific site of infection, allowing the local release of high concentrations of drug, while keeping the total dose of drug administered low. This high local dose is able to destroy the infecting fungi before they can develop resistance, thereby overcoming this worrisome issue and translating into fewer side effects upon the patient [104].

That being said, it is also important that the research done, not only focuses on formulating these systems, but also in overcoming the major challenges that their placing on the market faces: the physical instability of nanoparticles, their small capacity of drug loading, the cytotoxicity/immunogenicity, and the high cost of production and standardization, given the complexity of the formulations. Besides that, there is almost a complete lack of studies in vivo as reaching the therapeutic range needed to perform these studies has proven to be an arduous job. That lies in the fact that, in many cases, there is an anticipated release of the drug, aggregation and precipitation of the nanoparticles, and the accumulation in non-target tissues.

## 2. Nanotechnology and Mycology

**Figure 2:**
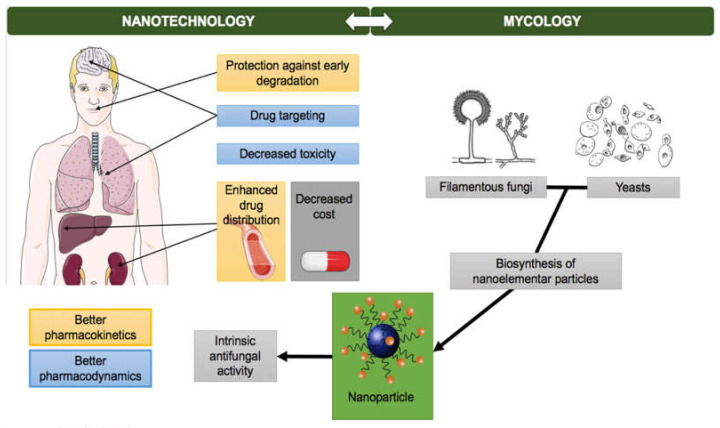
Bidirectional relationship of Nanoparticles and Mycology: nanotechnology has proven to be useful in improving antifungals pharmacokinetics and pharmacodynamics and many fungi have been used to biologically synthetize nanoparticles.

Mycology and Nanotechnology have created a bidirectional relationship throughout the years. This dynamic interface between mycology and nanotechnology led to the creation of the term “myconanotechnology” (Figure 2) [110]. Nanotechnology has proven to be an interesting strategy to increase the potency and efficiency of conventional antifungals, to enable a decrease in toxicity and cost, to avoid an anticipated degradation, to ameliorate the drug distribution, by increasing the circulation time and improving pharmacokinetics, and also to improve drug targeting, with promising in vitro and in vivo results [5]. Furthermore, many metallic nanoparticles have been used against human and plant pathogenic fungi in the light of their intrinsic antifungal activity and a wide spectrum of fungi are able to biosynthesize nanoelemental particles [110].

### 2.1. Antifungal Potential of Nanoparticles

Metallic nanoparticles have been used to eliminate fungi that are pathogenic to Man and to plants, because of their intrinsic antimicrobial activity [110]. The exact mechanisms this activity occurs through are only hypothesized and can be explained through three main pathways: (1) direct uptake of nanoparticles, (2) indirect activity of nanoparticles by production of reactive oxygen species (ROS), (3) impairment of cell wall/membrane through accumulation [111]. It is highly probable that it is the combination of these multiple pathways that is responsible for antimicrobial activity [112].

Nanoparticles undergo dissolution processes thanks to their electrochemical potential [113]. This leads to their separation into ions within the microbial fluid or in the culture medium. These ions also accumulate in the interior or exterior causing an inhibitory answer against microtubules. The accumulation of nanoparticles outside the microtubules causes the formation of layers that block cellular respiratory chain and destroy the microtubules [111].

The electrical charge of the nanoparticle is vital for the interaction that occurs between it and the carried drug. The electrostatic mechanism justifies why the antimicrobial activity was firstly described in silver nanoparticles. It is widely accepted that the positive charge of the silver ion is crucial for the antimicrobial activity of these nanoparticles through electrostatic attraction between the negatively charged cellular membrane of microorganisms and the positively charged membrane of nanoparticles [111]. Ag^+^ has high affinity to thiol groups in cysteine of respiratory chain enzymes, therefore it uncouples the synthesis of adenosine triphosphate (ATP). Ag^+^ also binds to proteins of transport from the respiratory chain, causing the leak out of protons and thus the collapse of the proton motive force. Furthermore, Ag^+^ obstructs the uptake of phosphate and so promotes the efflux of intracellular phosphate [113].

Silver nanoparticles exhibit potent antifungal activity against clinical isolates and ATCC strains of *Trichophyton mentagrophytes* with concentrations of 1–7 μg/mL and a MIC (minimum inhibitory concentration) of 25 μg/mL against *Candida albicans* [114]. Silver nanoparticles also reveal good antifungal activity against *Aspergillus niger*, by inhibiting spore germination and preventing biofilm formation; when combined with simvastatin, there is an additive and synergistic effect that increases the antifungal effect, perhaps because simvastatin, as an ergosterol synthesis inhibitor (see Table 1), disrupts fungal cell membrane, which allows the entry of the nanoparticles [115].

Metals present in nanoparticles can act as catalysts, reacting with biomolecules thanks to their high specific surface area, inducing the direct production of free radicals when exposed to the acidic environment of lysosomes or interacting with oxidative organelles [112,113].

ROS, like superoxide anions, hydroxyl radicals, and hydrogen peroxide, are oxygen-derived by-products formed when a material is exposed to an oxygenated environment, allowing their interaction with biomolecules. This way, they can cause an imbalance between the production of reactive species and the capacity of the biological system to detoxify reactive intermediates or repair damage [111]. Although the antioxidant cell defense prevents the effects of ROS to some extent, excessive ROS production may cause oxidative stress and lipid peroxidation, leading to membrane impairment, mitochondrial dysfunction, and DNA damage. That being a toxic mechanism to human cells, cytotoxicity tests should be performed on nanoparticles that owe their antimicrobial effects to ROS production, therefore avoiding interactions and toxic reactions in human beings [111].

Chitosan and its chemical derivates have been used as building blocks for drug delivery nanoformulations in light of their biocompatibility, biodegradability, and mucoadhesivity, presenting some advantages such as in situ gelling performance, mucoadhesive properties, and ability to prolong the release of low-molecular-weight compounds to macromolecular drugs [116]. Chitosan nanoparticles have proven to exhibit great antimicrobial activity against Candida infections [1]. According to the literature and research already undertaken, this antimicrobial activity is attributed to positively charged amino groups that react with negatively charged groups of lipopolysaccharides and proteins on the surface of the microbial cells, resulting in disintegration of the cell membrane. By doing this, the nanoparticles are able to bind with DNA molecules and inhibit mRNA and protein synthesis. In the specific case of fungi, chitosan acts by inhibiting the sporulation and germination of spores, by interfering with the activity of the growth-promoting enzymes [1,117].

Zinc oxide nanoparticles (ZnONPs) have also proved antifungal activity against dermatophyte infections and other pathogenic fungi, such as Candida and Aspergillus [1]. Meanwhile, the synergistic antifungal activity of ZnONPs was evaluated along with common antifungal drugs, which revealed that their inhibitory efficiency can be increased in combination with ZnONPs, which could possibly reduce the overuse of these drugs, decrease their toxicity, and increase their antifungal activity [118]. In addition, these nanoparticles might be an interesting and promising alternative to conventional preservatives in cosmetics in the future [119].

In addition to these nanoparticles mentioned above, dendrimers also exhibit antifungal activity and provide the opportunity for complex therapy in which dendrimers are both the drug carrier and the adjunctive component of the dosage form [41].

### 2.2. Synthesis of Nanoparticles by Fungi

Thanks to their tolerance and ability to bioaccumulate metals, fungi now occupy a central role in the biological production of metallic nanoparticles (Table 3) [10]. They can not only be used to produce the nanomaterials that will make up the nanosystem coating, but also be, themselves, carried by the nanosystem. This way, new ecological processes are developed, which imply a reduced waste of solvents and chemical substances. The biosynthesis methods are simpler and allow size and shape control of nanoparticles. Besides fungi, other organisms are used to synthesize nanoparticles, for instance, bacteria, plants, or plant extracts [110]. Compared to bacteria, fungi produce higher quantity of enzymes, which is translated into a higher yield in the nanoparticles production. Furthermore, their growth, both in laboratory and at an industrial scale, is easier to control [110].

The biological synthesis can either be intracellular or extracellular according to the nanoparticles’ locations. In intracellular synthesis, the ions are transported to the inner part of microbial cells to form nanoparticles in the presence of an enzyme. The nanoparticles formed inside the organism are of shorter size, when compared to the extracellular ones, because there is nucleation of particles inside the organisms. Extracellular synthesis has more applications than intracellular, as there is no need to join cellular components from the cell. The majority of the fungi produce nanoparticles extracellularly as a result of their secretory components that participate in nanoparticles reduction and capping [10].

Both yeasts and filamentous fungi can be used to synthetize nanoparticles. The process of synthetizing filamentous fungi-mediated nanoparticles is easy and cost-effective, since the mycelia in the biomass has high surface area and high intracellular metal absorption. Moreover, the fungi cell wall has many functional groups that facilitate the absorption to the metals [110].

Silver nanoparticles are the most fundamental among metallic nanoparticles that are involved in biomedical applications, specially cancer diagnosis and antimicrobial therapy [114]. One of the most remarkable examples is the extracellular synthesis of silver nanoparticles from filamentous fungi, like *Fusarium solani*, a pathogenic fungus isolated from infected onions. Ingle et al. [121] believe that, in these cases, the fungus does the “phagocytosis” of the nanoparticles, depositing them in their cellular wall, by binding to their functional groups. Afterwards, the fungus carries the nanoparticles and excretes them through exocytosis. The silver nanoparticles obtained were quite stable in solution because fungus-secreted proteins capped the nanoparticles. The authors argued that the procedure with this fungus could work for other metal nanomaterials such as gold and platinum with countless applications in the medical field [121].

Afterwards, many other scientists were able to optimize the biological production of silver nanoparticles using other fungi, such as *Fusarium oxysporum* [122], *Cochliobolus lunatus, Beauveria bassiana* [123], *Bipolaris maydis* [124], and many others. It was also concluded that some of these biologically synthesized silver nanoparticles showed enhanced antifungal activity with fluconazole against *Phoma glomerata, Phoma herbarum, Fusarium semitectum, Trichoderma* sp., *Phoma glomerata, Phoma herbarum,* and *Candida albicans* [114]. Surprisingly, it was additionally observed that the potential of silver nanoparticles is much wider than the inhibition of human and plant pathogenic fungi, as they also inhibit indoor fungal species such as *Penicillium brevicompactum, Aspergillus fumigatus, Cladosporium cladosporoides, Chaetomium globosum,* and *Stachybotrys chartarum* [125].

Likewise, yeasts can be useful for nanoparticle synthesis, as they produce enzymes responsible for the reduction of metallic salts and their conversion into elementary nanoparticles. Some examples are the biosynthesis of cadmium nanoparticles by *Candida glabrata* and *Schizosaccharomyces pombe* [110] and of selenium and silver nanocompounds by *Saccharomyces cerevisiae* in aerobic conditions [126,127].

### 2.3. Antifungal Drug Administration

The administration of antifungal drugs is not restricted to oral or parenteral routes. Other routes such as transungual, pulmonary, and ocular have also acquired great importance in the treatment of certain fungal infections. The development of a drug delivery system for attaining therapeutic concentration at these target organs is a challenge that requires a comprehensive understanding of the dynamics and specific features of the nails, lungs, and eyes, respectively.

#### 2.3.1. The Transungual Route

This special topical route acquires singular importance in antifungal therapy, mainly when onychomycosis (*Tinea ungium*) caused by dermatophytes (such as *Trichophyton rubrum*) or yeasts are concerned [128]. These infections affect the nail plate and/or the nail bed and are very frequent [129].

Drug delivery through nails has its own challenges and the use of nail penetration enhancers is compulsory in formulations. The nail plate is composed by cross linked keratin linkages, an extensive bonding network responsible for its rigidity. Although there has been considerable research regarding new approaches for transungual drug delivery, topical permeability was limited by its barrier properties. Therefore, the lookout for novel approaches is important to enhance treatment efficacy and reduce treatment time and relapse rate [128]. There are still no nanotech solutions for this purpose, but medicated nail lacquers loaded with ciclopirox or amorolfine are the most feasible delivery vehicles [130]. The main hurdle in the development of nail lacquers for nail disorders is delivering the therapeutically effective concentrations to the site of infection, which is often under the nail [129].

#### 2.3.2. Pulmonary Delivery

Drug delivery to the lungs is challenging because the availability of therapeutic quantities of antifungal drug at the site of infection is often inadequate on account of high blood flow turnover in the tissue. This can be problematic since a local and prolonged drug release is desirable when targeting this organ.

Developing a nanoparticle-based system and delivering it to the lungs in case of a pulmonary infection may help retain the antifungal in lungs over a prolonged period and may reduce the toxicity compared to the parenteral route [72,131].

A study in which voriconazole was encapsulated within a PLGA nanoparticle revealed that this is a better option to deliver the drug in deep lung tissue at a high concentration for a prolonged period of time and assumedly to provide greater antifungal effect in fungal infections. This was concluded because these nanoparticles were retained for a prolonged time in lungs and showed higher biodistribution when compared to voriconazole alone [72].

#### 2.3.3. The Ocular Route

The eye is a particularly puzzling organ for drug delivery systems. Physical barriers that hinder drug access into the eye limit the drug bioavailability. In addition, physiological processes like blinking and tear drainage reduce the residence time of ocular drug delivery systems and drastically reduce the amount of trans-corneal drug absorption [131,132].

Fungal ocular infections are much less prevalent when compared to bacteria or virus. However, fungal keratitis is considered the second cause of blindness in developing countries and is an important cause of morbidity [133,134]. People with prolonged use of corticosteroids or antibiotics, with diabetes mellitus, with corneal trauma or surgery, and even people wearing contact lenses are at a higher risk of having fungal keratitis [134].

New formulations such as polymer-based nanoparticles, liposomes, dendrimers, SLNs, spanlastics, and niosomes were developed to enhance drug bioavailability to the eye and to minimize antifungal adverse effects [135]. They also have the ability to overcome the disadvantages of conventional eye drops, like short residence time, by prolonging the contact time at the corneal surface and achieving a sustained release of the drug. In addition, the encapsulation into these systems protects antifungals from degradation promoted by the metabolic enzymes on conjunctival and corneal surfaces and in tear fluids [134].

A study assessed the efficacy of a fluconazole liposomal formulation for candidal keratitis that aimed at prolonging the antifungal action by increasing the contact time. The authors reported 86.4% healing observed in rabbits treated with fluconazole encapsulated liposomes, presumably because of higher viscosity and lipid solubility of fluconazole-loaded liposomes [136].

Furthermore, a fluconazole-loaded niosomal gel can also be successfully used as a topical drug delivery system for corneal fungal infections. Ethosomes are known to improve both transcorneal permeability and ocular bioavailability of poorly water-soluble drugs, which is a tremendous improvement in topical ocular drug delivery [137].

Spanlastics also enhance permeability and bioavailability of antifungal drugs and their nano-size makes them able to reach the posterior segment of the eye, when eye drops are used [138]. For instance, an itraconazole-loaded spanlastic proved to be safe and non-irritant to the eyes; moreover, the elasticity of these vesicles serves as a drug deliver for both anterior and posterior eye diseases [59].

### 2.4. An Overview of Nanoparticle Types and Their Applicability on Antifungal Therapy

#### 2.4.1. Lipid Nanoparticles

Albeit the safety and tolerability of systemic antifungal therapy has improved considerably, a rising proportion of immunocompromised patients are receiving systemic antifungal agents for progressively longer treatment courses and that increases the probability of longer-term risks, drug interaction, and other toxicity-related reactions in different organs [139].

Ambisome^®^ was the first successful example of a nanotech antifungal drug and it was manufactured by the Nexatar Company USA in 1990 [84,140]. In this formulation, amphotericin B was incorporated in an unilamellar liposomal bilayer of approximately 45–80 nm. Compared to the conventional formulation, this one showed less toxicity and prolonged circulation time, which suggests a higher distribution rate [84]. Even though Ambisome^®^ circumvented the initial toxicity issues, its daily-basis usage is still limited by its cost [5]. Thus, nanosomal formulations of amphotericin B were latter developed, with a special focus on lipid nanoparticles for intravenous delivery, which showed lower cytotoxicity against human kidney cells and much less hematotoxicity compared to the ones already marketed [141].

Besides amphotericin B, some other drugs have also been incorporated into liposomes with promising results. One good example of that is nystatin, a drug used mainly topically because of its poor oral bioavailability, thus excluded for the treatment of systemic fungal infections [134]. A daily injection of liposomal nystatin, however, showed great efficacy in the treatment of invasive aspergillosis, although with some mild renal toxicity and some infusion-related events [98]. Additionally, a multilamellar liposome gel system containing clotrimazole revealed to be useful in the treatment of vaginal candidiasis, providing a sustained release in the vaginal fluid and a reduced dosing interval [43].

Econazole is a topical antifungal that may be an irritant to the skin when conventional vehicles are employed. Applying a liposomal formulation has revealed to be a good strategy to minimize the irritating potential and to increase the compliance. Bioavailability studies of the liposomal gel revealed a seven-fold increase in the drug concentration in the epidermis, when compared to a control. Thus, the authors proved that it is possible to reduce the amount of drug applied and keep the therapeutic efficacy, minimizing the cutaneous irritation [142].

Transfersomes are generally regarded as an upgrade of liposomes, because they overcome their poor penetration through the stratum corneum, by modifications in the bilayer composition [134]. They are elastic nanovesicles made up from phospholipids and edge activators, most often surfactants or hydrophilic detergents with high mobility.

These particles are essentially studied as carriers for dermal and transdermal delivery, but have also shown to be effective carriers for genetic material and vaccines [143]. Ultradeformable liposomes or transfersomes were tested in a topical delivery system for amphotericin B and were found to enhance the drug penetration towards deep skin layers in a scale 40 times higher than Ambisome^®^, making this system clinically significant [93].

Ethosomes are essentially a type of transfersome that employs ethanol instead of the edge activator molecules; the main difference lies in the fact that ethanol evaporates once applied on the skin, whereas the edge activator molecules remain on the skin surface after water evaporation from the formulations [144].

Ethanol also plays an essential role in enhancing the delivery of both hydrophilic and lipophilic drugs without impairing their deformability and elastic characteristics, which was a significant disadvantage of transfersomes [143]. Henceforth ethosomes show great potential as topical delivery systems for antifungal drugs [134].

The formulation of an ethosomal gel containing econazole nitrate pointed out the outstanding potential of this system to function as a topical delivery system, since it allowed controlled drug release, increased antifungal activity, and good stability after storage [31].

Fluconazole is used both for local and systemic fungal infections. Despite its worldwide usage, it shows low penetration rate because of its high solubility and low permeability. The preparation of an ethosomal gel for topical delivery of fluconazole has proved to be an efficient way to overcome the issues associated with bioavailability, degradation, stability, and side effects of this therapeutic agent [67].

Although the great potential of ethosomes, when the concentration of ethanol used is above 30–40% w/w, the vesicular membrane tends to become more permeable and leaky, leaking out the entrapped drug, specially hydrophilic/ionized drugs [45,145]. Furthermore, this high ethanolic content may affect the skin causing irritation or contact dermatitis. To solve that, Cavamax W7, a permeation enhancer, was developed so as to improve topical delivery and to reduce the amount of ethanol used therefore reducing the risk of adverse effects [45]. The Cavamax W7 ethosomes were able to reach the last layer of epidermis (stratum basale) which turned them into a very valuable tool in the treatment of deep-seated fungal infections [134]. A clotrimazole encapsulated Cavamax W7 composite ethosome gel presented a more stable and more efficient vesicular system than the conventional ethosomal formulation along with a better antifungal activity against *Candida albicans* and *Aspergillus niger* [45].

Transethosomes are lipid vesicles with irregular shapes that represent a combination of the concepts applied to transfersomes and ethosomes, having a high content of ethanol (up to 30%) along with an edge activator molecule. This combination causes a rearrangement in the lipid bilayer of transethosomes, allowing them to have both higher deformability and skin permeation/penetration than the other deformable molecules. The formulation of these vesicles also presents the advantage of being quite easy to scale up, which constitutes an enormous advantage for industrial purposes [144]. Contrary to other deformable vesicles, transethosomes improve skin delivery of drugs both under occlusive and non-occlusive condition [146]. For these reasons, they are considered the most promising lipid nanoparticles in antifungal therapy.

An econazole nitrate loaded transethosome gel for transdermal delivery has been developed. Comparing it with the marketed cream of econazole nitrate, the authors found out that the former presented less ex vivo penetration, higher ex vivo skin retention, and higher in vitro antifungal activity [32]. Transethosomes containing terbinafine, amphotericin B, ketoconazole also disclosed enhanced permeation [146]. In addition, voriconazole transethosomes significantly enhanced skin permeation and deposition of the drug when compared to conventional liposomes, transfersomes, and ethosomes [74].

Regardless of the fact that transethosomes represent the state-of-the-art in ultradeformable vesicles, they still have some drawbacks that are important to mention such as the solubility that the drugs need to fulfil in both lipophilic and aqueous environment so that they can reach the dermal microcirculation and access the systemic circulation. In addition, the molecular size of the drug needs to be reasonable to make the percutaneous absorption possible (40–200 nm) [146].

Solid Lipid Nanoparticles (SLN) and Nanostructured Lipid Carriers (NLC) have become very attractive options to the development of drugs for cutaneous application and as an alternative to overcome the drawbacks of liposomes. They provide an occlusive effect by virtue of their ability to form a surface film that reduces the transepidermal water loss, which can reduce the atopic eczema symptoms and improve the skin appearance [147]. Besides that, they exhibit an excellent tolerability profile and its reduced size promotes a closer contact with the stratum corneum, intensifying the skin penetration of drugs. These particles are also able to increase the chemical stability of compounds sensitive to light, oxidation, and hydrolysis [148].

One application of SLN and NLC is the incorporation of azoles to treat cutaneous fungal infections. The most common azoles (clotrimazole, miconazole, econazole) are extremely water insoluble, being very difficult to administrate and to release those drugs in infection sites. However, compounds with lipophilic character can be efficiently encapsulated in SLNs [107].

In 2010, a formulation was developed in which the SLN nanoparticles were dispersed in a hydrogel to carry miconazole nitrate (MN), an azole frequently used in cutaneous fungal infections [149]. The studies indicated that this MN-loaded SLN-bearing hydrogel formulation was less prone to cause cutaneous irritation in comparison to the MN hydrogel and MN suspension formulation, showing an even greater efficacy in animal models (male albino rabbits). Furthermore, SLN-bearing hydrogel provided sustained release of miconazole (slow initial release with a lag time of 1 h and a sustained drug release over a 24 h period) with greater drug deposition on the skin. This happens because gels help disperse the matrix carriers evenly, increasing the contact time and the deposition of carriers on the skin, resulting in a higher cutaneous penetration of the drug [16,149].

#### 2.4.2. Polymeric Nanoparticles

Polymeric nanoparticles are produced either from natural or synthetic polymers, in which the drug is dissolved, trapped, or bound to the nanoparticle matrix. Depending upon the composition of the organic phase and the preparation method, nanocapsules (matrix-like structure) or nanospheres (core-shell morphology) can be obtained [110,150].

These nanoparticles are capable of carrying proteins, DNA, and drugs, such as antifungals, for cells and specific target organs, which increases the safety profile. Their nanometric size promotes an effective permeation through the cellular membranes and increases their stability in blood flow.

It is also important to point out the gelling systems that can be used to formulate polymeric carriers: microsponges and nanosponges, amphiphilic gels and emulgels or gellified emulsions, which play an important role in cutaneous drug delivery [143].

Microsponge and nanosponge systems are polymer-based spheres that can encapsulate or suspend many substances and then be incorporated into a dosage form (hydrogel) or be used for oral delivery [44,66,151].

A polymeric microsponge based gel system was successfully produced for the topical delivery of fluconazole, with especially great entrapment efficiency, production yield, and extended drug release, which allows a reduction in application frequency, a feature with great importance in recidivist and prolonged fungal infections [66].

A nanosponge based gel formulation loaded with clotrimazole showed controlled release with reduced side effects, indicating the safe and effective profile of these colloidal carriers for topical use [46]. An econazole nanosponge hydrogel was developed and was found to solubilize poorly soluble drugs and also had the ability of forming a local depot for sustained drug release [33].

Amphiphilic gels are semisolid systems of nonionic nature that can be used as topical/transdermal carriers without promoting irritation of the skin. They aim at delivering the antifungal in a level within the therapeutic window for as long as possible and to avoid fluctuations in plasma drug level [69].

An amphiphilogel of fluconazole was successfully formulated for topical application, presenting overall stability and cumulative drug release within the range expected [69].

Emulgels have dual release control system (emulsion/microemulsion and gel), which increases the overall stability of the antifungal formulations [143]. In comparison to conventional creams or ointments, they exhibit better application properties, faster, and more complete drug release profile and they lack greasiness and residues upon application [47].

Emulgels are suitable solutions to incorporate hydrophobic drugs in water soluble gel bases, for instance the incorporation of clotrimazole in an emulgel formulation prepared using either Carbopol 934 or HMPC 2910 showed good physical properties, stability, and antifungal activity [47].

A formulation with amphotericin B encapsulated in poly(lactic-co-glycolic acid) PLGA in association with ε-caprolactone was developed and proved the potential of polymeric nanoparticles in preventing cytotoxicity, yet preserving the fungicidal efficacy. With this polymeric combination, the authors described an amphotericin B encapsulation efficacy of 84% and a fungicidal effect for *Candida albicans* equal to the free drug, with the advantage of presenting less toxicity and less mortality associated [91].

A nanoformulation of the same drug in PLGA was also developed but, this time, it was functionalized with dimercaptosuccinic acid (DMSA). This acid exhibits tropism to lungs, being appropriate to be included in a formulation in which it is desired a release at this site. In this study an intraperitoneal administration was also tested and verified that the therapeutic effect on paracoccidioidomycosis (PCM) was equivalent to the free drug. The greatest advantage of this formulation was that it only required to be taken every three days, given the slow release of amphotericin B from the nanoparticles, and did not require a daily administration as the currently commercialized formulation [152].

Chitosan is a very versatile polymer owing to the ease presented by their groups to be modified or deacetylated [153]. Some studies have shown that this polymer also possesses antioxidant, antimicrobial, and anti-inflammatory properties, becoming very attractive from a biopharmaceutical viewpoint [154]. Some other studies pointed out that chitosan nanoparticles themselves have a huge potential to become safe and effective antifungal agents [155].

Chitosan nanoparticles containing amphotericin B were initially developed with the aim of avoiding the drug gastrointestinal degradation, increasing the stability and the bioavailability in target organs like the lung, the liver, and the spleen, whilst decreasing renal exposition. The researchers determined that when administered by an oral route, these nanoparticles were effective in treating visceral leishmaniosis, aspergillosis, and candidiasis, showing an efficacy comparable to the parenteral Ambisome^®^ formulation. Moreover, this natural polysaccharide was also employed in the cutaneous release of amphotericin B in wounds caused by infected burns with *Candida albicans*, promoting better tissue healing and increased antifungal activity, when compared to the conventional formulation [156].

Polymeric micelles present a unique architecture where the hydrophobic core can incorporate hydrophobic drugs, such as antifungals, which leads to a very significant improvement in their aqueous solubility [134]. These systems have attracted attention in drug delivery because their critical micelle concentration (CMC) is several 1000-fold lower (<10 mg/L) than classic micelles, this means that ionic surfactants have greater ease in self-assembling in water to form micelles [157].

Amphotericin B has been incorporated in polymeric micelles to treat brain fungal infections. The system was intended to improve drug solubility and permeability through biological membranes as well as obviate issues like high toxicity and low efficacy against *Candida meningoencephalitis* [94].

**Figure 3:**
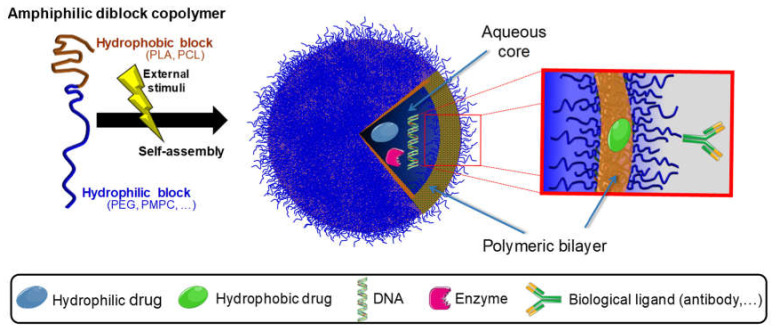
Schematic representation of the formation of a polymersome and its versatile properties. Polymersomes are generally self-assembled from block copolymers, presenting a unique structure that is able to encapsulate different biological molecules.

Polymersomes are spherical structures composed of an aqueous core surrounded by a polymeric bilayer membrane, being viewed as synthetic analogues to liposomes. They are very versatile structures, which spontaneously self-assemble from amphiphilic diblock copolymers. This capacity increases drug efficacy and enables them to encapsulate both hydrophilic and hydrophobic drugs, including antifungals [158,159]. Furthermore, polymersomes present stimuli-responsive drug release, which means that their physical and chemical properties are mutable in response to certain features (pH, temperature, redox conditions, light, magnetic field, ionic strength, or even concentration). This ability is promising in drug-controlled release, an important feature in antifungal drug therapy [159,160]. In Figure 3 the process by which a polymersome is formed is represented as well as its ability to carry different biological molecules.

A research group developed an amphotericin B loaded polymersome by solvent injection method, using (PEG)_3_-PLA as co-polymer. This formulation was compared with two marketed formulations (Fungizone^®^ and Ambisome^®^) in terms of release, molecular organization of amphotericin B, and hemolysis. The results were similar to the marketed formulations, which indicated the potential for further in vivo development [92].

Dendrimers are homogenous polymeric tridimensional nanoarchitectures characterized by a highly-branched and symmetrical structure consisting of a central core (a single atom or a group of atoms), building blocks of repeating units emanating from the core (generations), and a high density of water-soluble functional groups on the surface (terminal group) [161,162,163]. The elements are added through sequential interactive chemical reactions to the central core, the spaces within the voids facilitate the encapsulation of active substances within the dendritic structure, and the terminal functional groups dictate the efficacy of nucleic acid complexation or drug entrapment [164]. Their nano and uniform size structure (2–10 nm in diameter), high degree of branching, water solubility, multivalence, well-defined molecular weight, and available internal cavities makes them extremely valuable as drug delivery systems and as carrier systems for antifungal agents [161,165].

Albeit different types of dendrimers have been described, the most frequently used for antifungal therapy are polyamidoamine (PAMAM) and polypropylene imine (PPI) dendrimers [163]. PAMAM dendrimers containing ketoconazole were shown to improve the solubility and the in vitro release of the drug and also to enhance the antifungal activity of ketoconazole [41].

#### 2.4.3. Metallic Nanoparticles

Three main groups of metallic nanoparticles, gold, silver, and magnetic, can be used for the vectorization of antifungal drugs.

Metallic nanoparticles can be synthetized in three different ways: chemically, physically, and biologically. Chemical synthesis has been associated with many side effects related with the absorption of toxic chemical particles on the surface of nanoparticles [10], thereupon the biological synthesis is acquiring some expression [166].

Gold nanoparticles are used in immunochemical studies to identify protein interactions and in DNA fingerprinting to detect DNA in a sample. They are also used to detect aminoglycosides like streptomycin, gentamicin, and neomycin [10].

Silver nanoparticles are the most efficient on account of their antimicrobial efficacy against bacteria, viruses, and other eukaryotic microorganisms. In fact, they are the most used materials, being applied as antimicrobial agents in textile industry, for water treatment, in solar protectors, etc. [10].

Albeit nanostructures of iron, cobalt, and nickel exhibit superparamagnetic properties and high magnetic susceptibility, superparamagnetic iron oxide nanoparticles such as magnetite (Fe_3_O_4_), hematite (α-Fe_2_O_3_), and maghemite (γ-Fe_2_O_3_), are the most studied types of magnetic nanoparticles. This type of nanoparticle has received special attention by virtue of their capacity to be influenced by magnetic fields, therefore being easily directed and released in a specific site of the organism [10,167].

Superparamagnetic iron oxide nanoparticles present other unique properties, for instance: low toxicity, biocompatibility, potent magnetic targeting capacity, and chemical inertia, thus, they have many biomedical applications, for example in the cancer research field, steam cells, tissue repair, drug release, genetic therapy, DNA analysis, and clinical diagnosis through magnetic resonance [10,167].

Nowadays, nanoparticles of superparamagnetic iron oxide are the ones that answer more efficiently to an external magnetic field, being the ones with more potential to become drug carriers [5]. In order to bypass cytotoxic effects, these Fe_3_O_4_ magnetic nanoparticles need to be coated, usually with DMSA (meso-2,3-dimercaptosuccinic acid); this is a procedure that is important not only to increase cell internalization and biocompatibility, but also to carry active molecules to the nanoparticle’s surface, essential to drug delivery [168].

#### 2.4.4. Other Drug Delivery Systems

Niosomes are non-phospholipid vesicles made of non-ionic surfactants that serve as drug depots in the body as they release the drug in a sustained fashion through its bilayers. They are also able to improve oral bioavailability of poorly soluble drugs, enhance the skin permeability of topical drugs, protect the enclosed active substance from deleterious factors, and increase the stability of the entrapped drug [169].

Nistatin was successfully encapsulated in niosomes and a safe and effective formula for parenteral administration was obtained. This formulation provided reduced nephrotoxicity and hepatoxicity in female Wistar rats and showed pronounced efficacy against *Candida albicans* with a higher level of drug in vital organs [99]. The encapsulation of ciclopirox in niosomes increased the half-life, promoted a prolonged drug release, and minimized the side effects [103]. Given the poor water solubility of griseofulvin and its slow absorption from oral route, a niosome was produced in order to enhance bioavailability of this active substance, to accelerate its absorption and to obtain a sustained release, acting as a depot inside the body [100].

Ketoconazole niosomal gel showed more prolonged action than conventional ketoconazole formulations, hence it can be developed in order to improve the antifungal activity [38].

Spanlastics are termed as modified niosomes which present better permeability because they have edge activators in their composition, like transfersomes and transethosomes [134,145].

These systems show great flexibility allowing them to pass through fenestrations smaller than their own radius in order to enter the cell, simultaneously minimizing the possibility of damaging the vesicles while squashing [138].

Firstly, there were ketoconazole-loaded spanlastics for ocular drug delivery [40] and then spanlastics were loaded with terbinafine hydrochloride for treatment of onychomycosis [79].

Microemulsions are colloidal carriers that consist of a liquid dispersion of oil and water stabilized by an interfacial film of surfactant. Due to this, they are able to incorporate drugs of different lipophilicity [170]. They are promising colloidal carriers because of their transparency, ease of preparation, and long-term stability [171].

When it comes to drug delivery through stratum corneum, microemulsions are very resourceful considering the ability of the oils and surfactants included in their composition to act as skin penetration enhancers [170,172]. Furthermore, they are also good candidates for oral delivery of poorly water-soluble drugs as they can improve their solubilization.

Terbinafine hydrochloride is an example of a slightly water-soluble drug, which presented higher solubility, an increase in the dissolution rate, and better efficacy when incorporated within a microemulsion [170,171]. Likewise, a microemulsion system of voriconazole also showed a significant increase in the antifungal activity against *Candida albicans*, along with better drug skin penetration [173].

Nanoemulsions are thermodynamically and kinetically more stable than emulsions, so they have been evaluated as colloidal carriers to improve the efficacy and tolerability of some antifungal drugs. The capacity of nanoemulsions to dissolve large quantities of drugs with low solubility, their compatibility, their ability to protect drugs from enzymatic degradation and hydrolysis as well as their capacity to penetrate the deeper skin layers, turns them into ideal drug delivery vectors [143,174].

A nystatin nanoemulsion for topical application was developed and was found to have a higher antifungal effect than nystatin itself, representing a therapeutic improvement [97].

Silicon dioxide nanoparticles have been studied as drug carriers to enhance the antimicrobial activity of some drugs, because of their biodegradability, low toxicity, capacity to stimulate macrophages, and the ease by which they are synthetized and modified. The foremost advantage of these nanoparticles is that they can be loaded with large amounts of drug [95,175].

There are four main types of silica nanoparticles, but their antifungal potential is yet to be studied in some cases:

- Nitric oxide-silica nanoparticles with proven anti-biofilm activity [108];
- Metal modified silica nanoparticles, which can include silver or copper, metals that have a very well documented antimicrobial effect, derived from the cell membrane and DNA damages, interaction with enzymes from thiol groups or are associated with generating hydrogen peroxide [176];
- Surface-modified silica nanoparticles by quaternary ammonium compounds loaded with antifungal agents [177,178];
- Bioglasses and bioceramics [179].

Amongst them, mesoporous silica nanoparticles are the ones who have become the most promising candidates for many biomedical applications, specifically for antifungal therapy, because of their uniformed mesoporous tunnels and narrow pore size distribution. Besides that, they present outstanding biocompatibility and chemical stability and can be degraded and metabolized over a relatively short term. These aspects allow high drug loading and reduce the probability of particle-induced toxicity [95,180].

In a study, one group of mice with candidiasis induced by *Candida albicans* was treated with silica nanoparticles functionalized with amphotericin B and the other group was treated with officinal amphotericin B. It was possible to observe that the first group showed a significant increase in survival rates [95]. This confirms the fact that silica nanoparticles coated with quaternary ammonium surfactants have higher fungicidal and fungistatic effect against *Candida albicans* than colloidal silver [177,178].

In order to increase the oral bioavailability of itraconazole, a poorly water-soluble antifungal drug, mesoporous silica particles formulation was developed. It was concluded that ordered mesoporous silica can, in fact, be considered a promising carrier seeing that it enhances the oral bioavailability of extremely low water-soluble drugs [60].

It has been demonstrated that metals can easily accumulate in soil and then enter the food chain, thus the combination of Ag+ ions with silica prevents the formation of these ions and reduces its toxicity [176]. That is the reason why silica nanoparticles have also been studied for agricultural purposes as safe and effective alternative fungicides, for example to manage tomato early blight [180].

## 3. Hidden Potential and Challenges of Natural Antifungal Compounds

From 1981 to 2014, 32 new chemical entities were placed on the market to treat fungal infections: one from a biological source (interferon gamma-n1), that is a peptide produced by a biotechnological procedure; three derived from a natural product that suffered a semisynthetic modification (anidulafungin, caspofungin, micafungin); 25 totally synthetic discovered by random screening or through the modification of an existing agent (fluconazole, itraconazole, ketoconazole, amorolfine, ciclopirox; three synthetically synthesized with the molecule or the pharmacophore mimicking a natural product (butenafine, liranaftate, terbinafine) [181].

In fact, almost 90% of the antifungal agents approved within this period were from a synthetic origin. This paucity of natural products in modern treatments remains a reality and 1950s agents like amphotericin and griseofulvin are still widely used [181].

One good example of an efficient employment of natural products to nanoparticle synthesis happened with *Mentha pulegium* L., commonly known as pennyroyal, a flowering herb with antitussive, carminative, and antiseptic effects. This herb was successfully used to synthesize stable colloidal silver nanoparticles with promising antifungal effects against *Candida albicans* [182].

On the other hand, the marine environment represents a valuable and unexplored platform to the discovery of new compounds [183]. In Table 4, there is an overview of the natural products that have shown in vitro or in vivo potential as antifungal agents isolated from diverse marine organisms: microorganisms (bacteria and fungi), invertebrates (sponges, corals and sea cucumbers), and marine algae are presented.

Sponges have always interested pharmacologists, chemists, and biologists as a rich source of antimicrobial compounds with peculiar activities. These colonial organisms have a sessile nature, hence the necessity of producing compounds as a way of protecting themselves, communicating, or modulating their cellular functions [184].

Peptides have high relevance as potential drugs, given their large spectrum of bioactivity. Sponges that belong to the Theonella gender are a recognized source of uncommon peptides with antibacterial, antifungal, and anti-HIV properties. The antifungal activity of some of these peptides is even surplus to other commercial formulations, as seen through their diffusion zone in the agar diffusion method [184].

Although some formulations containing marine compounds are already being subjected to clinical trials, none of them target fungal infections [183].

Theopapuamide A is a cyclic depsipeptide (a peptide in which one or more amides were replaced by an ester group) being firstly isolated from the sponge *Theonella swinhoei* from Papua New Guinea [183]. Its isolation, structural elucidation, and stereochemical analysis are well described in literature [185]. It is capable of inhibiting the growth of *Candida albicans* wild strains and also strains resistant to amphotericin B [186]. In addition, it presents anti-HIV activity, thanks to the 3,4-dimethyl-L-glutamine residue in its chemical structure [183,184].

Besides the marine environment, a considerable number of studies have been conducted on medicinal plants and alternative compounds, such as secondary metabolites, phenolic compounds, essential oils, and extracts. Some plant extracts (*Curcuma zedoaria*, *Psidium guajava*, *Plectranthus amboinicus*, *Lippia alba*) have shown activity against *Candida* spp. and others (*Asteraceae*, *Euphorbiaceae*, *Rubiaceae,* and *Solanaceae*) against filamentous fungi. Despite the streamline nature of the discovery process, the biological activity found during the screening of plant extracts, may not be experimentally reproducible. This can be due to many reasons: the chemical constituents in the crude extracts may be different, the solvent used to extract may destroy some compounds and chemical composition may vary according to the growth stage or geographic origin. Some components of essential oils, mainly terpenes or terpenoids, such as eugenol, camphor, curcumin, geraniol, and linalol show activity against a wide variety of Candida species and others like clemateol or citral are active against *Trichophyton* spp. or *Malassezia* spp. respectively. Propolis, which represents the resinous substances collected from plants by bees, demonstrated activity against *Candida* spp., some dermatophytes, and onychomycosis. Ajoene, a compound derived from garlic, has shown effectiveness in the treatment of paracoccidioidomycosis and *Fusarium* spp. infections. Other compounds such as saponins, alkaloids, flavonoids, coumarins, xanthones, lignans, and tannins also presented antifungal activity [187].

There is, in fact, a growing awareness of the limited structural diversity in existing compound libraries and that there are many benefits in exploiting the huge chemical diversity and high biological activity of natural compounds. Furthermore, it is possible to use these natural compounds as platforms to discover drug-like characteristics and produce new substances [188]. Therefore, natural compounds with antifungal properties represent a valuable alternative to current antifungals and their incorporation in nanocarriers is a significant step ahead in nanoformulation, besides being a more sustainable option, because it employs marine resources [183].

Since the discovery of penicillin, the pharmaceutical industry has extensively relied on natural products as sources of structural templates for drug discovery and development [188,189].

Regardless of this fact, the discovery and development of natural-derived medicines has been on a steady decline over the last years, possibly due to high throughput screening. This tool has changed the paradigm of drug discovery in the pharmaceutical industry since it allows the automatic screening of thousands to millions of drug candidates within vast libraries. There is every likelihood that these libraries contain a significant representation of all existing compounds, complicating the discovery of a new natural chemical entity itself [190].

Although a valuable and precious resource, natural products brought about their fair share of challenges in a wide variety of features, compromising their inclusion in the clinical pipeline.

The main issue lies in the fact that natural substances are not always abundant and they generally require laborious extraction and purification steps through complex, expensive, and time-consuming processes, often to obtain just few quantities of extract [190]. Moreover, there are no standard protocols and research groups modify the existing procedures, making the studies not reproducible and not comparable [187].

In addition, environmental aspects pose significant hurdles for drug discovery and development because the substance may come from an endangered species and its overexploitation can lead to habitat destruction [188].

The regulatory requirements for substances containing natural substances (medicines, nutraceuticals, and cosmeceuticals) range from rather strict to non-existent and vary between regions, being under the surveillance of different authorities [188].

## 4. Ongoing Clinical Trials on Myconanotechnology

Several pharmaceutical industries and authors have been performing clinical trials on antifungal nanomedicines. Despite the growing improvement on applying nanotechnology for drug development, just a few of those nanomedicines have been approved for their clinical applications. The major issues with clinical trials are their time-consuming process and the difficulty while accessing and researching information on previous nanomedicine trends. In the mycology field, amphotericin-B is the active substance that represents the majority of the nanomedicines already available in the market: Amphotec^®^ (Three Rivers Pharmaceuticals, approved in 1992), Abelcet^®^ (Sigma-Tau, approved in 1995) and Ambisome^®^ (Gilead Sciences, approved in 1997), all lipid-based formulations [191,192]. An econazole liposomal formulation (Pevaryllipogel^®^) is also available on the market [193]. In Table 5 some examples of recent clinical trials are presented.

## 5. Conclusions

The present pace of antifungal drug development is highly unlikely to keep up with the clinical needs, especially with the uprising of resistance to current agents. Therefore, more therapeutic answers to fungal illnesses are urgently needed.

The subject of sustainability and environmental impact has gained considerable relevance in today’s societies and scientific research, motivating a heated debate on whether there should be an active research for new natural compounds with biological activity or if the pillar of today’s research for new compounds should be high throughput screening. The fact is that two out of the three major classes of antifungal drugs (polyenes and echinocandins) were screened from natural products. The utilization of natural compounds, such as chitosan is, irrefutably, a more sustainable option and brings both environmental and pharmacokinetic advantages to nanotechnological approaches. Compounds from a marine origin exhibit an undeniable potential, but their activity and toxicity mechanisms are yet to be clarified.

Nanoparticles have been presented as promising solutions, mainly due to their ability to target specific sites where fungi are harbored, their capacity to enhance the pharmacological effect of drugs, optimizing their physiochemical characteristics, thereby allowing an administration through a more comfortable route. All these features can enable lower dosing, more comfortable regimens, increased bioavailability, and less serious adverse effects.

Special attention has been given to certain kinds of nanoparticles nowadays mainly because of the outstanding features they exhibit: (a) magnetic nanoparticles and their capacity to directly restrict fungal growth, (b) ultradeformable vesicles (transethosomes) and their ease on scaling up, (c) mesoporous silica nanoparticles and their high drug loading, (d) polymersomes and their ability to carry both hydrophobic and hydrophilic substances and to respond to external stimuli, (e) PAMAM dendrimers and their versatile and biocompatible structure. We speculate that future antifungal therapies will mostly lie in these five types of nanoparticles and will take advantage of the current knowledge of using them for other purposes (for instance, as anticancer agents).

These new nanotechnological systems should be able to surpass the issues already mentioned and should also mean a significant upgrade when comparing to the conventional treatments, fighting antifungal resistance, presenting a broad spectrum of activity, with an emphasis on potency increase and little host toxicity, thus having the potential to be industrially produced.

Interdisciplinary cooperation may be the key to a striving success of nanomedicine and nanotechnology, not to mention a proper exploitation of natural medicinal products. Physicists, health-care researchers, and clinical researchers have complementary knowledge that could be put together (for example, innovative screening strategies and novel chemical libraries) so that more effective, practical, and safe nanoparticles are designed. That would increase the chances of an industry funding and, ultimately, a rethinking of current antifungal arsenal, while providing a better quality of life for patients.

## Figures and Tables

**Table 1:** Targets of each group of antifungals [6,7].

Class	Target (Mechanism of Action)	Antifungal	
Azoles	Ergosterol (inhibition of lanosterol 14-α-demethylase)	Imidazoles	Miconazole
			Econazole
			Ketoconazole
			Clotrimazole
		Triazoles	Itraconazole
			Fluconazole
			Voriconazole
Allylamines	Ergosterol (inhibition of squalene epoxidase)	Terbinafine	
		Naftifine	
		Butenafine	
Polyenes	Cell membrane (production of ROS)	Amphotericin B	
	Ergosterol (inhibition of lanosterol 14-α-demethylase)	Nystatin	
Echinocandines	Cell wall (block of β-1,3 glucan synthesis)	Caspofungin, Micafungin, Anidulafungin	
Other antifungals	Chelation of polyvalent metal cations	Ciclopirox	
	Microtubules (prevention of the formation of the mitotic spindle)	Griseofulvin	
	Ergosterol (inhibition of D14 reductase and D7-D8 isomerase)	Amorolfine	

**Table 2:** Some of the novel drug delivery systems already developed for each antifungal drug.

Antifungal Drugs	Novel Drug Delivery Systems	Routes of Administration	Dosage Forms	References
Miconazole	Niosomes	Transdermal	Gel	[18]
	SLN	Oral	N.A.	[19]
		Topical	Gel	[20]
	Microemulsion	Topical	N.A.	[21]
	Liposomes	Topical	Gel	[22]
	Nanoemulsion	Topical	N.A.	[23]
	Nanosponges	Vaginal	Gel	[24]
	Transfersomes	Topical	Gel	[25]
Econazole	Microemulsion	Percutaneous	N.A.	[26]
		Topical	Gel	[27]
	SLN	Topical	Gel	[28]
	NLC	Topical	Gel	[29]
	Liposomes	Topical	Gel	[30]
	Ethosomes	Topical	Gel	[31]
	Transethosomes	Transdermal	Gel	[32]
	Nanosponges	Topical	Hydrogel	[33]
	Niosomes	Transdermal	Gel	[34]
	Polymeric micelles	Topical	N.A.	[35]
	Nanoemulsion	Topical	N.A.	[36]
Ketoconazole	SLN/NLC	Topical	Gel	[37]
	Niosomes	Topical	Gel	[38]
	Microemulsion	Oral	N.A.	[39]
	Spanlastics	Ocular	N.A.	[40]
	Dendrimers	Topical	Hydrogel	[41]
	Liposomes	Topical	N.A.	[42]
Clotrimazole	Liposomes	Topical	Gel	[43]
	Nanosponges	Topical	Hydrogel	[44]
	Ethosomes	Topical	Gel	[45]
	Niosomes	Topical	Gel	[46]
	Polymeric emulgel	Topical	Gel	[47]
	Polymeric micelles	Topical	N.A.	[35]
	SLN/NLC	Topical	N.A.	[48]
	Microemulsion	Buccal	Gel	[49]
		Vaginal	Gel	[50]
	Transfersomes	Transdermal/Topical	N.A.	[51]
Itraconazole	Transfersomes	Transdermal	N.A.	[52]
	SLN	Ocular	N.A.	[53]
	NLC	Inhalation	N.A.	[54]
	Niosomes	Topical	N.A.	[55]
	Microemulsion	Transdermal	N.A.	[56]
	Liposomes	Topical	N.A.	[57]
	Polymeric nanoparticles	Oral	N.A.	[58]
	Polymersome	Intravenous	N.A.	[54]
	Spanlastics	Ocular	N.A.	[59]
	Silica nanoparticles	Oral	N.A.	[60]
Fluconazole	Microemulsion	Vaginal	Gel	[61]
	Niosomes	Ocular	Gel	[62]
	Liposomes	Intravitral	N.A.	[63]
	SLN	Topical	Gel	[64]
	NLC	Oral	N.A.	[65]
	Microsponges	Topical	Gel	[66]
	Ethosomes	Topical	Gel	[67]
	Spanlastics	Ocular	N.A.	[68]
	Polymeric amphiphilogel	Topical	Gel	[69]
	Polymeric micelles	Topical	N.A.	[35]
Voriconazole	Microemulsion	Ocular	N.A.	[70]
	Polymeric nanoparticles	Ocular	N.A.	[71]
		Pulmonar	N.A.	[72]
	SLN	Topical	Gel	[73]
	Transethosome	Topical	N.A.	[74]
	Ethosome	Topical	N.A.	[75]
Terbinafine	Liposomes	Topical	Gel	[76]
	SLN	Topical	N.A.	[77]
	Transfersomes	Topical	N.A.	[78]
	Spanlastics	Transungual	N.A.	[79]
	Polymeric chitosan nanoparticles	Topical	Hydrogel	[80]
Naftifine	Microemulsion	Topical	N.A.	[81]
	Niosomes	Topical	Gel	[82]
Butenafine	Microemulsion	Topical	Hydrogel	[83]
Amphotericin B	Liposomes	Intravenous	N.A.	[84]
	SLN/NLC	Oral	N.A.	[85]
		Topical	N.A.	[86]
	Magnetic nanoparticles	Nasal instilation	N.A.	[87]
	Nanoemulsion	Topical	N.A.	[88]
	Polymeric nanoparticles	Intravenous	N.A.	[89]
		Oral	N.A.	[90]
	Polymersomes	Oral	N.A.	[91,92]
	Transfersomes	Topical	N.A.	[93]
	Micelles	Intravenous	N.A.	[94]
	Silica nanoparticles	Intravenous	N.A.	[95]
Nystatin	SLN	Topical	N.A.	[96]
	Nanoemulsion	Topical	N.A.	[97]
	Liposomes	Intravenous	N.A.	[98]
	Niosomes	Parenteral	N.A.	[99]
Griseofulvin	Niosomes	Oral	N.A.	[100]
Ciclopirox	Niosomes	Topical	Gel	[101]
Caspofungin, Micafungin, Anidulafungin, Amorolfine	No nano-tech studies yet released			

N.A.: the dosage form is not mentioned in the reference cited; SLN: Solid Lipid Nanoparticles; NLC: Nanostructured Lipid Carriers.

**Table 3:** Some examples of metallic nanoparticles produced by fungi and their method of synthesis [10,120].

Fungal Species	Nanoparticles Type	Method of Synthesis
*Phoma* sp.	Silver	Extracellular
*Fusarium oxysporum*	Gold; Magnetite	Extracellular
*Verticillium* sp.	Silver	Intracellular
*Aspergillus fumigatus*	Silver	Extracellular
*Aspergillus niger*	Silver	Extracellular
*Fusarium semitectum*	Silver	Extracellular
*Trichoderma asperellum*	Silver	Extracellular
*Phaenerochaete chrysosporium*	Silver	Extracellular

**Table 4:** Overview of antifungal natural compounds produced by marine organisms [183,184].

Marine Organism	Source Organism	Type of Compound	Compound Name	Spectrum of Activity
Bacteria (30% of total)	*Bacillus licheniformis*	Glycolipid	Ledoglucomide C, Iedoglycolipid	*Aspergillus niger, Rhizoctonia solani, Botrytis cinerea, and Colletotrichum acutatum, Candida albicans*
	*Bacillus subtilis*	Lipopeptide	Gageopeptides A-D	*R. solani, P. capsici, B. cinerea, C. acutatum*
	*Actinoalloteichus sp. NPS702*	Macrolide	Neomaclafungins A-I	*Trichophyton mentagrophytes*
	*Streptomyces sp.*	Peptide	Mohangamide A	*C. albicans*
	*Bacillus marinus*	Macrolide	Macrolactins T and B	*Pyricularia oryzae, A. solani*
	*Tolypothrix*	Lipopeptide	Hassallidin A	*A. fumigatus and C. albicans*
	*Chondromyces pediculatus*	Peptide	Pedein A	*Rhodotorula glutinis*
Fungi (15% of total)	*Stagonosporopsis cucurbitacearum*	Alkaloid	Didymellamide A	*C. neoformans, C. albicans, C. glabrata*
	*Aspergillus sclerotiorum*	Peptide	Sclerotide B	*C. albicans*
	*Penicillium bilaiae MA-267*	Sesquiterpene	Penicibilaenes A and B	*C. gloeosporioides*
Sponge (35%)	*Theonella swinhoei*	Peptide	Theonegramide, Theonellamide G, Cyclolithistide A	*C. albicans*
	*Halichondria cylindrata*	Peptide	Halicylindramide D and E	*Mortierella ramanniana*
	*Siliquariaspongia mirabilis, Theonella swinhoei*	Peptide	Theopapuamide A; B and C	*C. albicans*
	*Jaspis johnstoni*	Peptide	Jasplakinolide	*C. albicans, C. pseidrotropicalis, C. parapsilosis*
	*Monanchora arbuscular*	Alkaloid	Batzelladine L	*A. flavus*
	*Xestospongia muta*	Furan	Mutafuran D	*Cryptococcus neoformans var.grubii*
Corals (5%)	*Clavelina oblonga*	Alkanol	(2S,3R)-2-aminododecan-3-ol	*C. albicans ATCC 10231, C. glabrata*
Sea cucumbers (6%)	*Stichopus variegates*	Triterpene glycoside	Variegatuside D	*C. albicans, C. pseudo- tropicalis, C. parapsilosis, and M. gypseum*
Algae (9%)	*Caulerpa racemos*	Xylene	Caulerprenylol B	*T. rubrum*

**Table 5:** Some examples of ongoing clinical trials on myconanotechnology [194].

Trade Name/Sponsor	ClinicalTrials.gov Identifier	Antifungal	Nanoformulation	Clinical Phase	Disease
Sara Botros, Minia University	NCT04110834	Itraconazole	Nanoemulsion gel	II	*Tinea versicolor*
Sara Botros, Minia University	NCT04110860	Voriconazol	Nanoemulsion gel	II	*Tinea versicolor*
Matinas BioPharma	NCT02971007	Amphotericin B	Cochleate lipid-crystal nanoparticle	II	Vulvovaginal candidiasis
Matinas BioPharma	NCT02629419	Amphotericin B	Cochleate lipid-crystal nanoparticle	II	Mucocutaneous candidiasis
Ahmed Abdellatif, Al-Azhar University	NCT03752424	-	Silver nanoparticle gel	I	Mycosis
Mona Badran, Cairo University	NCT03666195	-	Titanium dioxide nanoparticles	Recruiting	Candidiasis
Rasha Hamed, Assiut University	NCT04431804	-	Silver nanoparticle	Recruiting	Invasive aspergillosis
Celtic Pharma Development Services	NCT01145807	Terbinafine (TDT067)	Transfersome	III	Onychomycosis
